# Family Predictors of Specialty Mental Health Service Use in Adolescents: A Prospective Cohort Study

**DOI:** 10.1007/s10802-025-01411-0

**Published:** 2026-01-07

**Authors:** Mina Moeineslam, Silje Steinsbekk, Lars Wichstrøm, Věra Skalická

**Affiliations:** 1 Department of Psychology, Norwegian University of Science and Technology, Trondheim, Norway; 2 Department of Child and Adolescent Psychiatry, St. Olav’s Hospital, Trondheim, Norway

**Keywords:** Service use, Specialty mental health service use, Adolescents, Mental health problems

## Abstract

One in seven adolescents has a mental disorder, accounting for 15% of the disease burden in this group. Identifying factors that are associated with adolescent help-seeking is essential for designing effective and sustainable mental healthcare. Family dynamics—particularly parental roles—are key determinants; however, these associations may evolve as adolescents become more independent. The present study aimed to identify family predictors of adolescents’ use of specialized mental health services and to examine potential age effects. We addressed these aims by analyzing four waves of biennially collected data (ages 12–18) from two birth cohorts in Trondheim, Norway (*n* = 822), utilizing clinical interviews and questionnaires, and accounting for potential confounders. The results of an autoregressive cross-lagged panel model revealed that, across ages 12–18, adolescents’ use of specialized mental health services at one time point consistently predicted continued use two years later. However, family functioning, parents’ perception of social support, and interparental conflict did not predict service use when controlling for multiple potential confounders (including prior service use, gender, parental occupation, cohabitation status, parental depression and anxiety, perceived need for help, indicators of adolescents’ emotional and behavioral problems, impairment, and stressful life events). The present findings indicate no evidence of a prospective association between these family factors and adolescents’ receipt of mental-health services. Future research should investigate which specific factors drive help-seeking and how these interact within different service systems to support more sustainable and equitable access to mental health care.

**Supplementary Information:**

The online version contains supplementary material available at 10.1007/s10802-025-01411-0.

Estimates from global studies indicate that approximately one in seven adolescents aged 10–19 is affected by a mental disorder, contributing to around 15% of the global disease burden within this age group (World Health Organization (WHO), 2021). In Norway, self-reported mental health symptoms among adolescents increased by 17% for girls and 5% for boys between 1992 and 2019, with an additional increase noted among girls during the COVID-19 pandemic (Potrebny et al., 2024). However, adolescents at highest risk often underutilize mental health services, while those experiencing lower distress increasingly seek care (Potrebny et al., 2021). This imbalance strains resources, restricts timely access to interventions, and undermines the sustainability of mental health services (Brownlee et al., 2017; Hansen et al., 2021). Adolescent help-seeking is not solely an individual process; it is deeply embedded within their broader social environment. While young people gain greater independence during this period, families continue to play a central role in shaping access to care (Ryan et al., 2014). Parents and caregivers are often the first to notice signs of mental health problems, make decisions about seeking professional help, and provide the practical and emotional support necessary for adolescents to engage with mental health services (Platell et al., 2023). Nevertheless, research on adolescent mental health service use has predominantly focused on individual need factors, with comparatively less attention paid to family dynamics or the home environment. Patterns of communication, emotional support, conflict, and overall functioning within families may be associated with whether mental health needs are acknowledged, how decisions about help-seeking are made, and whether adolescents ultimately access services (Logan & King, 2001). Studying the family context, therefore, offers a critical opportunity to identify factors that shape service access and can inform the development of more effective early intervention strategies (Ryan et al., 2014). However, the role of family dynamics in adolescent help-seeking remains insufficiently understood. Additional research is needed to clarify how specific aspects of the home environment support or hinder care-seeking, thereby enabling more targeted and family-responsive mental health services. The present study addresses this gap by examining whether family factors predict the use of specialized mental health services within a birth cohort of Norwegian adolescents assessed at ages 12, 14, 16, and 18 years.

## Predictors of Mental Health Service Use in Adolescence - Theoretical Models and Current Empirical Evidence

The Behavioral Model of Health Care Utilization (Andersen, 1968) is the most commonly employed framework for examining factors that predict healthcare use. According to this model, three clusters of factors can influence healthcare utilization: predisposing, enabling, and need factors. Predisposing factors include demographic characteristics, social structure elements (e.g., education level, occupation, or family size), and individual beliefs (e.g., attitudes, values, and knowledge about health and healthcare services). Enabling factors reflect the resources available within families and communities that support access to healthcare, such as family resources, social relationships, and support systems. Lastly, need factors refer to the potential demand for health services, including perceived health status and evaluated health conditions (e.g., diagnosed by a healthcare professional) (Andersen & Newman, 1973). Models of service use for children and adolescents build on Andersen’s framework by incorporating the unique role of family dynamics. These adaptations emphasize the role of parents as gatekeepers; they are typically among the first to recognize their child’s distress, assess the need for professional help, and navigate the referral and access process (Stiffman et al., 2004). Incorporating family-level features such as parental involvement ensures that the model remains relevant when examining help-seeking behaviors in younger populations. Aligned with Andersen’s conceptualization of enabling factors, Ryan et al. (2014) identified aspects of the family environment and functioning as “family enabling factors,” recognizing the family’s role in providing social and instrumental support that facilitates adolescents’ access to mental health services.

### Family Enabling Factors

While family dynamics are increasingly recognized as important enabling factors in adolescent mental health service use, findings remain inconsistent. Some studies have found that higher levels of family support may act as an informal resource, thereby reducing the utilization of formal services (LeCloux et al., 2016), whereas another study showed that supportive father behavior can encourage adolescents to seek help (Reeb & Conger, 2011). Maiuolo et al. (2019) found that adolescents’ perceptions of parental authoritativeness and support enhanced the intention to seek help but were unrelated to actual service utilization. Conversely, increased parental overcontrol has been associated with a lower likelihood of adolescents’ service use (Ryan et al., 2014). Reigstad et al. (2006) reported that family functioning was the strongest predictor of adolescent referral to specialty mental health services. Furthermore, while one study linked parent’s perception of social support to greater adolescent service use (Bussing et al., 2015), another found that socially isolated parents may also seek more services (Martinez & Lau, 2011).

The primary motivation for seeking help is typically to alleviate the child’s mental health problems and associated impairment—that is, to reduce their level of need. However, beneficial treatment outcomes may also positively impact parents and the broader family context. To clearly distinguish between the outcomes of help-seeking and the factors that predict it, prospective studies are essential. Yet, the majority of existing research has been cross-sectional (Bussing et al., 2015; Douma et al., 2006; Langeveld et al., 2010; Lindsey et al., 2010; Reigstad et al., 2006; Staghezza-Jaramillo et al., 1995; Zwaanswijk et al., 2003), and longitudinal studies have typically featured short follow-up periods, spanning only a few weeks to a couple of years (LeCloux et al., 2016; Maiuolo et al., 2019; Reeb & Conger, 2011; Ryan et al., 2014; Turner et al., 2007). This may limit the ability to assess how family factors relate to adolescents’ mental health service utilization over time. Understanding these associations is important, as family factors may shape not only whether adolescents initially seek help, but also whether they continue engaging with services and thus experience sustained benefits (Maiuolo et al., 2019). Given the increasing autonomy that comes with age, the role of family dynamics is likely to vary across different stages of adolescence. However, existing research has not adequately tested the role of family functioning in adolescents’ mental health service use throughout adolescence. While some previous longitudinal studies have included family-related variables, they typically examined only one or two enabling factors (LeCloux et al., 2016; Maiuolo et al., 2019; Martinez & Lau, 2011; Reeb & Conger, 2011). Apart from Ryan et al. (2014), little attention has been given to a broader range of family factors. To address this limitation, the present study adopts a more comprehensive approach by examining three key family enabling factors: family functioning, parent’s perception of social support, and the quality of parental relationships. These family enabling factors are considered important because they capture critical dimensions of the home environment, such as communication patterns, emotional bonds, and supportive networks, all of which shape adolescents’ coping strategies and their willingness to seek professional help (Romić & Ljubetić, 2021; Zimmer-Gembeck & Locke, 2007). For example, it has been shown that clear, supportive family communication that emphasizes the adolescents’ strengths and resources helps adolescents feel understood, thus increasing their willingness to consider professional help (Boulter & Rickwood, 2013; Moen & Hall-Lord, 2019). Adolescents from dysfunctional families might lack the necessary support or encouragement to seek professional care (Martyn et al., 2014; Rickwood et al., 2007). In families characterized by high levels of conflict, teens might feel their mental health struggles only exacerbate family tensions, leading them to avoid seeking help due to concerns it might worsen the situation (Greenwald O’Brien et al., 2023). Furthermore, caregivers who receive affirmational support—that is, validation of their parenting decisions—are more likely to seek mental health services for their adolescents, indicating that supportive networks can reduce stigma and promote healthcare use (Bussing et al., 2015). In sum, a positive family environment characterized by high family functioning and robust social support is expected to foster adaptive coping mechanisms and encourage the use of mental health services, whereas interparental conflict may have the opposite effect. Family dynamics are particularly relevant in the context of adolescents with more severe mental health conditions, who typically require specialty mental health services providing high-intensity care. Given the critical need to ensure access for this high-risk group, the present study focuses on the associations between family dynamics and adolescents’ use of specialized services.

### Predisposing and Need Factors

In line with Andersen’s model, and to ensure a comprehensive approach, the present study adjusts for predisposing and need-related factors, which are well-established predictors of adolescent service use. These factors are consistently associated with service utilization and are therefore included as covariates to isolate the unique contribution of family-related influences. Research has identified several consistent predictors, including gender (Burnett-Zeigler & Lyons, 2010; Fisher et al., 2018; Guo et al., 2015; Zwaanswijk et al., 2003), with females typically using services more than males; family socioeconomic status (SES), with studies reporting associations with both higher (Amone-P’Olak et al., 2010) and lower SES (Cheung et al., 2009; Palacio-Vieira et al., 2013; Reiß et al., 2021; Vu et al., 2018); parental divorce (Reijneveld et al., 2014); and parental mental health problems (Breland et al., 2014). Among need-related factors, adolescents’ mental health problems (Laitinen-Krispijn et al., 1999; Splett et al., 2018), functional impairment (Ezpeleta et al., 2002; Wu et al., 2001), parental perception of need (Brattfjell et al., 2021; Kim & Kim, 2022), and exposure to stressful life events (Ezpeleta et al., 2002; McChesney et al., 2015) have all been shown to predict adolescents’ use of mental health services. Critically, predisposing and need-related factors may themselves shape family dynamics. For example, research has shown that lower SES (Li et al., 2014; Mansfield et al., 2013), parental mental illness (Marston et al., 2018; Wiegand-Grefe et al., 2019), adolescents’ mental health difficulties (Baena et al., 2021; Jiménez et al., 2019), and exposure to stressful life events (Shimizu et al., 2023; Stanek et al., 2020) are associated with lower family functioning and cohesion. Accordingly, this study adjusts for these factors to isolate the unique contribution of family enabling factors.

## The Current Study

**Fig. 1:**
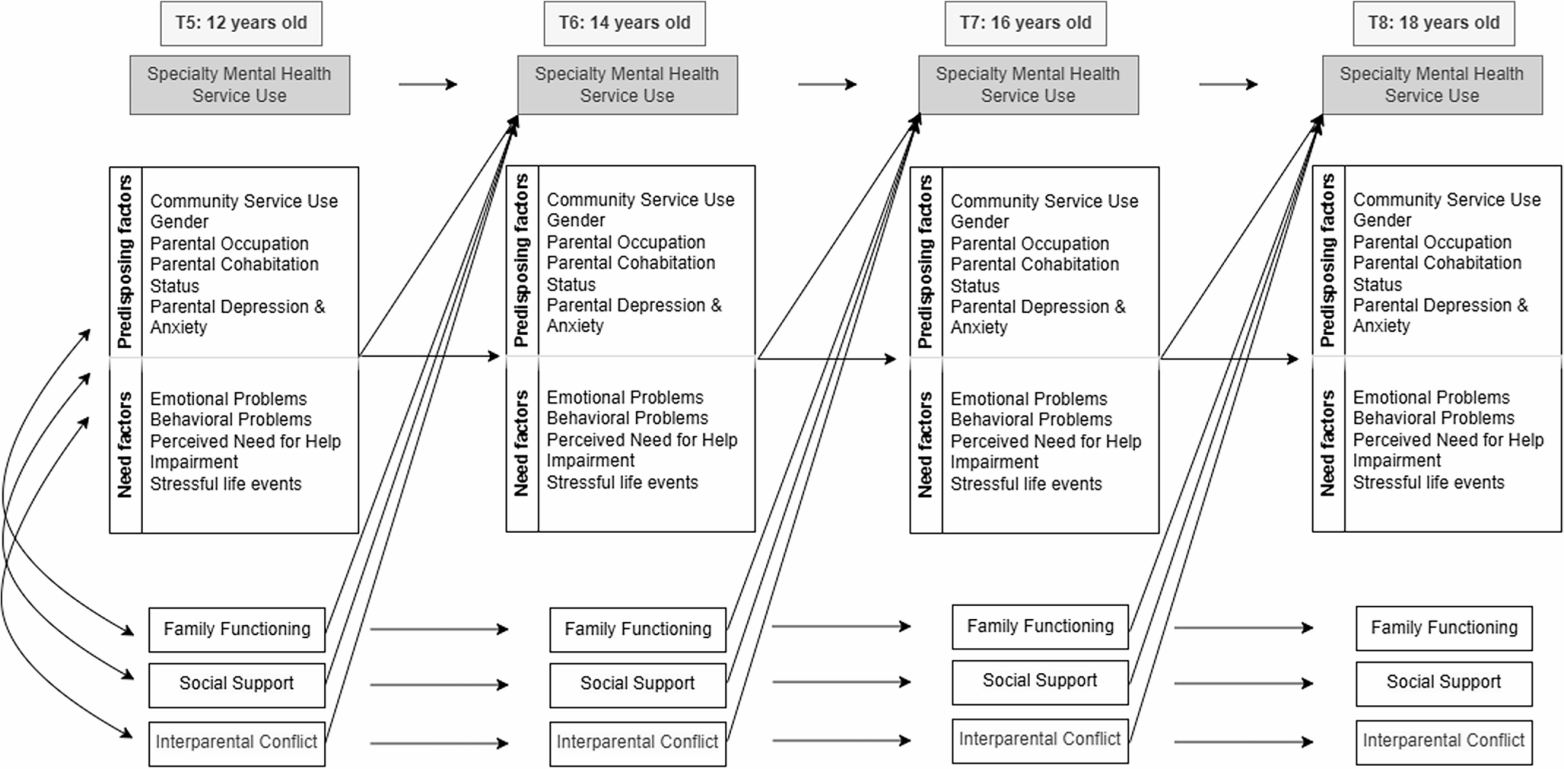
Conceptual framework of predictors of specialty mental health service use. Note. Conceptual framework illustrating predictors of specialty mental health service use at four time points. The model includes family enabling factors (family functioning, social support and interparental conflict), and a propensity score of predisposing and need factors, their autoregressive paths, and cross-lagged paths linking these factors to service use. The cross-sectional correlations between family factors, predisposing and need factors at T6, T7, and T8 were omitted from the figure for the reason of clarity

Informed by the Behavioral Model of Health Care Utilization and existing evidence, we hypothesized that positive family dynamics—specifically higher parent-reported family functioning and social support in parental role—will predict adolescents’ use of specialized mental health services two years later, accounting for a range of predisposing and need factors. Conversely, we expected that family conflict and aggression will predict a lower likelihood of subsequent service use. The following factors were controlled for: Predisposing: Previous community mental health service use, gender, parental occupation, parental cohabitation status, and parental depression and anxiety; Need factors: Symptoms of emotional and behavioral disorders, perceived need for help, impairment, and stressful life events. These covariates were incorporated as propensity scores, as illustrated in Fig. 1.

## Methods

### Participants and Procedure

We used four waves of data (at ages 12, 14, 16, and 18) from the Trondheim Early Secure Study (TESS; Steinsbekk & Wichstrøm, 2018). In TESS, all children born in 2003 and 2004 who were living in Trondheim, and their parents were invited to participate. In an invitation letter sent shortly before each child’s fourth birthday, parents received the Strengths and Difficulties Questionnaire (SDQ; Goodman, 2001), and were asked to bring the completed questionnaire to the routine 4-year health check-up. The SDQ is a validated screen for emotional and behavioral problems in children aged 2–17. At the check-up, parents received detailed information about the study from the health care nurse and provided written informed consent. The study protocol was approved by the Regional Committees for Medical and Health Research Ethics, Mid-Norway.

As shown in the online supplementary materials (Online Resource 1, Figure S1), of the 3,016 children invited to participate, *N* = 2,477 (82.2%) provided consent. Parents who lacked sufficient Norwegian proficiency to complete the questionnaires and interviews were excluded (*n* = 176). To improve statistical power, children were stratified into four groups based on their SDQ scores (0–4, 5–8, 9–11, and 12–40); the probability of selection rose with higher scores—37%, 48%, 70%, and 89%, respectively. This oversampling of children with behavioral and emotional problems was accounted for in the analyses. At the first assessment, *N* = 1,007 children attended the university clinic with a parent (50.9% female; M = 4.40 years, SD = 0.18). Among accompanying parents, 84.8% were female, 93.0% were of Norwegian origin, 56.3% were married, 5.7% held leadership positions, and 72.1% possessed a college or university degree. Comparison with Statistics Norway data indicated that parental education levels were comparable to those of all parents of four-year-olds in Trondheim (Statistics Norway, 2012). Biennial follow-ups were conducted from ages 4 to 18: 6 years (T2, *n* = 802), 8 years (T3, *n* = 704), 10 years (T4, *n* = 703), 12 years (T5, *n* = 666), 14 years (T6, *n* = 636), 16 years (T7, *n* = 665), and 18 years (T8, *n* = 631). The present study focuses on adolescence (ages 12–18) and the analytical sample includes 822 participants who provided data at one or more of these waves.

Attrition at age 12 was higher among females than males (OR = 1.03, 95% CI [1.01, 1.05], *p* =.011). At age 14, greater emotional problems predicted lower odds of dropping out (OR = 0.98, 95% CI [0.97, 0.99], *p* <.001), whereas higher behavioral problems were associated with increased attrition (OR = 1.10, 95% CI [1.01, 1.19], *p* =.021); attrition was also higher among females (OR = 1.03, 95% CI [1.01, 1.06], *p* =.006). Attrition at age 16 was lower for females (OR = 0.55, 95% CI [0.39, 0.78], *p* <.001). By age 18, gender was no longer a significant predictor of attrition. Little’s MCAR test indicated that the data were not missing completely at random, χ²(5169) = 9073.79, *p* <.001; however, the normed chi-square value (χ²/df = 1.76) suggested only a moderate deviation from MCAR.

### Setting

The Norwegian health-care system comprises two tiers. Primary care—provided by general practitioners (GPs), community services, and social services—covers first-line needs, whereas secondary care consists of specialty services that typically require a GP referral (Saunes, 2020). Child-protection services and licensed psychologists can also refer children to specialty mental health services. All health-care services are free of charge for individuals younger than 18 years.

### Measures

#### Mental Health Service Use

##### Specialty Mental Health Service Use

The Child and Adolescent Services Assessment (CASA; Ascher et al., 1996) was used to assess mental health service use. The parent-report version of the CASA was administered at every wave; beginning at age 16, the adolescent self-report version was also administered. The CASA is a structured interview that captures children’s recent use of services for emotional, behavioral, learning, attentional, or substance-use problems. Informants reported the number of treatment sessions received since the previous assessment (i.e., two years earlier). From age 16 onwards, both adolescents and their parents completed the CASA interview. For the present study, a joint score combining information from both informants was used to create a dichotomous variable indicating whether the adolescent had attended at least one specialty mental health session during that period; participants were therefore classified as service users or non-users. Prior research indicates that both the parent- and self-report versions of the CASA demonstrate strong test–retest reliability (Farmer et al., 1994; Hoagwood et al., 2000; Horwitz et al., 2001).

#### Enabling Family Factors

##### Social Support in the Parental Role

Social support was measured with a parent-report questionnaire that was developed specifically for this study. What was of theoretical interest was the support that parents receive in their role of providing help to their offspring — that is, support in the parental role. As no scales exist that capture this construct directly, we drew inspiration from the well-validated Social Support Questionnaire of Sarason et al. (1983) and adapted it to assess social support in the parental role. At age 12, the scale comprised 12 items covering practical, informational, emotional, and acceptance-related support; at later waves (T6–T8), it was reduced to 8 items by removing practical help. Mean scores were based on all available items at each wave. For each dimension, parents rated both the availability of support and their satisfaction with it on a 7-point scale (1 = “no support at all” to 7 = “very high degree of support”; e.g., *“To what extent do you have someone who shows that they fully accept and respect you as a parent?”*), and a mean total score was used in analyses (α = 0.88 to 0.91).

##### Family Functioning

Family functioning was assessed with the 12‑item General Functioning subscale of the Family Assessment Device (FAD; Epstein et al., 1983). This subscale comprises 12 items—six positively worded and six negatively worded—rated on a 4-point Likert scale (1 = “strongly agree” to 4 = “strongly disagree”). The positively worded items (e.g., “We are able to make decisions about how to solve problems.”) were reverse-scored so that lower total scores reflect poorer global family functioning (α = 0.86 to 0.88).

##### Interparental Conflict

Interparental conflict was measured with the Conflict and Problem-Solving Scales (CPS; Kerig, 1996). At each wave, the participating parent reported on both their own and their partner’s interparental conflict behavior (e.g., throws things, slams doors, breaks things). Behaviors were rated on a 4‑point scale (1 = “never”, 2 = “seldom”, 3 = “sometimes”, 4 = “often”). Self- and partner ratings were summed. We then combined scores across five CPS subscales—Avoidance/Capitulation (10 items), Stonewalling (7 items), Verbal Aggression (6 items), Physical Aggression (9 items), and Child Involvement (5 items). Higher total scores indicate a greater prevalence of negative conflict behaviors (α = 0.88 to 0.95).

#### Predisposing Factors

##### Community Health Service Use

Data on community-based service use were obtained with the CASA (Ascher et al., 1996). At each wave, parents reported the number of treatment sessions their child had received since the previous assessment (i.e., 2 years earlier); beginning at age 16, adolescents provided parallel self-reports. Consistent with the specialty-care variable, we used a joint score combining parent and adolescent reports to create a dichotomous indicator denoting whether the participant had attended at least one community mental health session during the preceding two-year interval. Participants who reported any contact were coded as service users, whereas those with no contact were coded as non-users.

##### Parental Depression and Anxiety

Parental symptoms of depression and anxiety were assessed with the Hopkins Symptom Checklist–25 (HSCL-25; Mollica et al., 1987), using a 24-item version of the HSCL-25 (Derogatis et al., 1974). The HSCL‑25 is a self‑report questionnaire consisting of 10 anxiety items and 14 depression items (e.g., “Have you had thoughts about taking your own life?”). Parents rated how much they had been bothered by each symptom on a 4‑point scale (1 = “not at all”, 2 = “a little”, 3 = “quite a bit”, 4 = “extremely”). A total symptom score was calculated by summing across all items, with higher scores indicating more severe emotional distress. The instrument has demonstrated satisfactory validity and reliability for detecting emotional distress in adults (Derogatis et al., 1974; Glass et al., 1978), and displayed good internal consistency in the present sample (α = 0.89–0.92).

##### Parental Occupation

The parent who attended the university clinic reported both their own and their partner’s occupational status, which was coded according to the International Labour Organization’s (ILO) six-level classification scheme (International Labour Office, 1990) ranging from unskilled worker to senior leader. We used the highest parental occupation score in the family.

##### Cohabitation Status

At each assessment, the participating parent reported any changes in marital or cohabitation status since the previous wave. For the present study, cases were coded based on whether the parents were living together or not.

#### Need Factors

##### Emotional and behavioral problems

At every wave, parents and adolescents were interviewed separately with semi-structured clinical instruments; a symptom was coded as present if reported by either informant. The Child and Adolescent Psychiatric Assessment (CAPA) was administered at ages 12 and 14, the Kiddie Schedule for Affective Disorders and Schizophrenia (K-SADS) at age 16, and the Structured Clinical Interview for DSM-5 Disorders–Present Version (SCID-5) at age 18. All interviews assess symptom onset, duration, and intensity according to the Diagnostic and Statistical Manual of Mental Disorders (DSM-5; American Psychiatric Association, 2013). Emotional problems included major depressive disorder (MDD), dysthymia, generalized anxiety disorder, social anxiety disorder, specific phobia, and separation anxiety disorder. Interrater reliability (intraclass correlation coefficients, ICCs) for blinded ratings of 15% of sessions was as follows: CAPA—MDD = 0.87, dysthymia = 0.85, anxiety disorders = 0.86; K-SADS—MDD = 0.81, dysthymia = 0.76, anxiety disorders = 0.94; SCID-5—MDD = 0.94, dysthymia = 0.87, anxiety disorders = 0.94. Separation anxiety disorder was not assessed at age 18, as the diagnosis is rarely applied in late adolescence. Behavioral problems encompassed oppositional defiant disorder (ODD), conduct disorder (CD), and attention-deficit/hyperactivity disorder (ADHD) at ages 12–16. At age 18, only ADHD was assessed, because ODD and CD are typically not diagnosed beyond early adolescence. Interrater reliability for behavioral disorders was high: CAPA—ODD = 0.90, CD = 0.85, ADHD = 0.90; K-SADS—ODD = 0.92, CD = 0.84, ADHD = 0.95; SCID-5—ADHD = 0.92.

##### Impairment

Psychiatric symptom impairment was evaluated using data from the CAPA and K-SADS, and SCID-5 assessments. If a symptom was present, its potential impact was assessed following the World Health Organization’s International Classification of Functioning, Disability, and Health (ICF; WHO, 2007). These impairment ratings were summed to create an overall impairment score.

##### Perceived Need for Help

Perceived need for professional assistance was assessed within the CAPA, K-SADS, and SCID-5 interviews using a semi-structured format. Interviewers first posed open-ended questions about behavioral, emotional, social, and attentional difficulties and then followed up as required to clarify whether the child had already received—or was perceived to need—professional help. Interviewers were instructed to probe thoroughly until a clear determination could be made. For example, parents were asked whether any of the discussed areas constituted a problem for their child and, if so, what type of help they believed was necessary. Responses were coded as reflecting a perceived need when at least one of 20 predefined problem areas (e.g., separation anxiety, depression, conduct problems, attention difficulties, or regulation issues) was endorsed.

##### Stressful Life Events

The CAPA and K-SADS include items covering 16 serious negative life events that may have occurred during the previous two years (e.g., death of a close adult, serious motor-vehicle accident, severe burns, near-drowning, major falls, witnessing serious violence or death, or experiencing physical or sexual abuse). In the CAPA, only parents reported these events; in the K-SADS and SCID, both adolescents and parents provided information. An event was coded as present if either informant endorsed it. The total life-events score at each wave equaled the sum of all endorsed events, with higher scores indicating greater exposure to stressful experiences.

### Statistical Analysis

Mplus (Version 8.1; Muthén & Muthén, 2017) was used for all analyses. Missing data were handled using full information maximum likelihood (FIML), which incorporates all available observations directly in the estimation rather than imputing values. FIML was applied under the assumption that data were missing at random (MAR) and is recommended in the methodological literature as one of the most appropriate approaches for handling missing data, performing comparably to multiple imputation (Enders, 2022). It is used under the assumption that data are missing at random (MAR). To account for the study’s intentional oversampling of children with emotional and behavioral problems, population weights, calculated by dividing the total number of eligible children by the number of participants in each SDQ stratum, were applied, resulting in population-representative estimates.

#### Preliminary analyses

Changes in family factors over time were evaluated with Wald χ² tests that constrained the means of each factor to equality across waves; a significant test indicated that at least one wave mean differed from the others.

To adjust for potential confounders without inflating model complexity or reducing statistical power, we carried out a two-step propensity-score analysis (Austin, 2011). First, we fit a logistic-regression model with specialty mental-health service use as the dependent variable and the selected confounders as predictors—namely, predisposing factors (previous community mental-health service use, gender, parental occupation, parental cohabitation status, and parental depression and anxiety) and need factors (emotional and behavioral problems, perceived need for help, impairment, and stressful life events). The predicted probability of service use from this model constituted each participant’s propensity score. In the second step, we entered the propensity score as a covariate, alongside the family-enabling variables, in the main analyses.

#### Main analyses

To examine the prospective associations between the family-enabling factors—family functioning, social support, and interparental conflict—and adolescents’ subsequent use of specialized mental-health services, we estimated an autoregressive cross-lagged panel model (ACPM) spanning four waves. The model included each participant’s propensity score for specialty-service referral as a covariate. It comprised two sets of paths: (a) autoregressive paths, which captured temporal stability by regressing each variable at Wave t on its own value at Wave t − 1, and (b) cross-lagged paths, which assessed prospective associations between family factors and service use (and vice versa) while controlling for baseline service use two years earlier. Concurrent correlations among all predictors were freely estimated at each wave. To identify the best-fitting and most parsimonious representation of the data, we compared a series of nested path models that imposed increasingly restrictive equality constraints (Table 3). Constraints that did not significantly degrade model fit were retained, thereby maximizing degrees of freedom.

## Results

Table 1 presents descriptive statistics for all study variables. The proportion of adolescents who used specialty mental-health services increased steadily across adolescence. Mean scores of each family factor—family functioning, social support, and interparental conflict—declined slightly over time. Wald χ² tests confirmed that these changes were statistically significant (family functioning: χ²(3) = 36.14, *p* <.001; social support: χ²(3) = 64.62, *p* <.001; interparental conflict: χ²(3) = 10.18, *p* =.017). Bivariate correlations between the family factors and specialty-service use appear in the online supplementary materials (Online Resource 1, Table S1).

**Table 1:** Descriptive statistics for predictors and outcome variable (*N* = 822)

Age	12	14	16	18
Specialty service use* (%)	3.80%	5.20%	10.20%	11.60%
Community service use* (%)	9.40%	11.20%	13.10%	10.80%
Stressful life events (M, SD)	0.41 (0.68)	0.50 (0.8)	0.44 (0.75)	0.41 (0.56)
Emotional problems (M, SD)	2.66 (3.33)	2.63 (3.95)	2.05 (3.79)	4.83 (7.06)
Behavioral problems (M, SD)	2.33 (3.18)	2.17 (3.18)	1.55 (3.46)	2.07 (3.67)
Impairment (M, SD)	1.35 (2.87)	1.45 (3.28)	1.90 (3.57)	0.77 (1.37)
Perceived need for help* (%)	10.20%	9.40%	5.50%	10.20%
Parental depression/anxiety (M, SD)	29.73 (6.73)	30.62 (7.18)	31.30 (7.23)	31.47 (8.20)
Parental occupation (M, SD)	4.59 (0.98)	3.59 (2.20)	4.64 (1.05)	4.61 (1.06)
Parental cohabitation status* (%)	24.4	27.2	29.6	28.9
Family functioning (M, SD)	3.39 (0.39)	3.37 (0.39)	3.30 (0.39)	3.32 (0.40)
Social support (M, SD)	5.49 (0.80)	5.22 (0.90)	5.13 (0.94)	5.15 (1.02)
Interparental conflict (M, SD)	16.53 (2.87)	16.33 (2.80)	16.41 (2.98)	16.19 (3.23)

*M* mean, *SD* standard deviation

*Asterisked variables represent the percentage (%) of participants who answered “Yes” for the respective variable

First, an unconstrained model (M1) was estimated, allowing all paths between the predictors and the outcome to vary freely across waves. In Model 2 (M2), the autoregressive paths for service use were constrained to be equal across time, and this restriction did not significantly degrade model fit relative to M1 (see Table 2), indicating stable autoregressive effects (i.e., the strength of the autoregressions were not age-dependent). In Model 3 (M3), both the autoregressive and the cross-lagged paths for service use and the propensity score were constrained across waves; model fit again remained unchanged from M2. In addition to these constraints, Model 4 (M4) also included constraints on family functioning (autoregressive and cross-lagged), which produced a significant decline in fit. Thus, Model 4 was rejected. Building on M3, we added constraints on the autoregressive and cross-lagged paths for parental social support (M5) and subsequently for interparental conflict (M6). In both models, these additional constraints did not significantly reduce model fit, suggesting that the effects of these family factors remained stable across time. Because family functioning at age 14 significantly predicted service use at age 16 in M6, we tested an additional model (M7) that forced this cross-lagged path to be equal across all intervals to test if age effects were evident. The resulting χ² difference test was non-significant, and the predictive path from family functioning to later service use lost statistical significance. Thus, Model 7 (M7) constitutes our final model, and the results are displayed in Table 3.

**Table 2:** Stepwise constrained path model of service use

Model	χ2	df	Scaling Factor	S-B χ2	df	*p*	Comparison
Unconstrained (Baseline) (M1)	116.73	87	1.19				
Service use paths constrained (M2)	114.85	89	1.24	1.04	2	0.59	M2 vs. M1
Service use & propensity score constrained (M3)	115.23	93	1.31	2.95	4	0.56	M3 vs. M2
Service use, family functioning & propensity score constrained (M4)	143.01	97	1.31	25.52	4	< 0.001	M4 vs. M3
Service use, propensity score & social support constrained (M5)	120.21	97	1.30	4.98	4	0.28	M5 vs. M4
Service use, propensity score, social support & interparental conflict constrained (M6)	126.84	101	1.32	6.24	4	0.18	M6 vs. M5
Family functioning constrained across ages (M7)	130.46	103	1.32	3.82	2	0.14	M7 vs. M6

At each step, both autoregressive and cross-lagged paths were constrained to be equal across waves. In the final model (M7), however, only the cross-lagged path for family functioning was constrained to assess age-related differences, with autoregressive paths freely estimated

χ² = chi-square, df = degrees of freedom, S–B χ² = Satorra–Bentler scaled chi-square

**Table 3:** Cross-Lagged model predicting specialty service use in the preceding two years

	Service use (14 years)			Service use (16 years)			Service use (18 years)		
	β	95% CI	*p*	β	95% CI	*p*	β	95% CI	*p*
Previous service use	0.26	(0.15, 0.37)	< 0.001	0.22	(0.14, 0.31)	< 0.001	0.30	(0.19, 0.41)	< 0.001
Propensity score as covariate	0.14	(0.06, 0.21)	< 0.001	0.14	(0.07, 0.21)	< 0.001	0.20	(0.09, 0.30)	< 0.001
Family functioning	0.05	(–0.00, 0.11)	0.06	0.04	(0.00, 0.08)	0.07	0.04	(0.00, 0.08)	0.06
Social support	− 0.03	(−0.08, 0.03)	0.31	− 0.02	(−0.06, 0.02)	0.31	− 0.02	(−0.06, 0.02)	0.31
Interparental conflict	0.05	(−0.01, 0.11)	0.13	0.04	(−0.01, 0.08)	0.13	0.04	(−0.01, 0.08)	0.13

Each column presents results from regression analyses predicting service use at ages 14, 16, and 18 years, respectively. β = estimated standardized regression coefficient; CI = confidence interval; p = probability value

All models included previous service use and propensity score as covariates

The model M7 fitted the data well (CFI = 0.989, TLI = 0.981, RMSEA = 0.018, 90% CI [0.005, 0.027], χ²(103) = 130.46, *p* =.035). As shown, prior specialty service use and the propensity score significantly predicted more mental-health service use across all three time intervals, although the effects were small. Previous service use: age 12 to 14: β = 0.26, *p* <.001; 14 to 16: β = 0.22, *p* <.001; and 16 to 18: β = 0.30, *p* <.001. The propensity score also showed significant effects across these intervals: age 12 to 14: β = 0.14, *p* <.001; 14 to 16: β = 0.14, *p* <.001; and 16 to 18: β = 0.20, *p* <.001. None of the family-related factors were significant. The stability of predictors across waves ranged from small to moderate: propensity score (β = 0.27–0.38), family functioning (β = 0.28–0.62), social support (β = 0.35–0.37), and interparental conflict (β = 0.57–0.66), all ps < 0.001 (Online Resource 1, Table S2). Within-wave covariances (Online Resource 1, Table S3) indicated that concurrent service use was negatively related to social support at age 12 (Cov = − 0.11, *p* =.016). At ages 14, 16, and 18, the covariances between service use and social support were not statistically significant. No significant concurrent covariances were observed between service use and family functioning or interparental conflict at any wave.

## Discussion

Theoretical models and prior research posit that features of the family environment can either promote or impede adolescents’ engagement with mental-health services. Most existing studies, however, are cross-sectional or short-term, limiting our understanding of whether these “family-enabling” factors predict service use across the full span of adolescence or vary by developmental stage. Using biennial data from a Norwegian birth cohort followed from ages 12 to 18, we tested whether family-level enabling factors prospectively predicted adolescents’ use of specialty mental-health services after adjusting for predisposing and need-related covariates. Contrary to what we expected, none of the family factors emerged as significant predictors. Thus, within the cultural context where this study took place, family functioning, perceived social support, and couple-relationship quality do not appear to play a central role in facilitating—or hindering—adolescents’ access to specialized mental-health care.

Our findings contrast prospective studies showing that positive family dynamics encouraged help-seeking (LeCloux et al., 2016; Reeb & Conger, 2011) and a study showing that greater social support predicted lower service use (Martinez & Lau, 2011). These discrepancies may reflect differences in sample characteristics, service systems, and study design— LeCloux et al. (2016) focused on suicidal adolescents, while Reeb and Conger (2011) examined a small, rural U.S. sample. However, our results are consistent with studies reporting no association between family factors and service use (Maiuolo et al., 2019). Our findings also partially align with those of Ryan et al. (2014), who observed no links between several family-climate variables and service use, although parental overcontrol was associated with reduced utilization. Several explanations may account for the present null effects. First, Norway’s readily accessible and cost-free mental-health services for adolescents may diminish the importance of family factors in predicting help-seeking. In health-care systems with limited access or high user fees, family resources can determine whether symptoms are recognised, interpreted as requiring professional care, and acted on (Sousa & Rodrigues, 2009). By contrast, within Norway’s welfare system, help-seeking follows a structured pathway: symptom detection, decision to seek care, GP consultation, referral to specialist services, and triage based on national priority guidelines (Norwegian Directorate of Health, 2024). This system prioritises clinical need—such as symptom severity and impairment—and does not depend on family functioning. Family dynamics may aid in recognising symptoms and initiating a GP visit, but once primary care is involved, access to specialty services is largely governed by clinical assessments and the national guidelines. This structure may explain why clinical need, rather than family context, emerged as the stronger predictor of specialist service use, and the significant effect of the propensity score further supports this interpretation. Second, as shown in the bivariate correlations (Online Resource 1, Table S1), the measured family-level variables were not significantly associated with adolescents’ use of mental-health services, though social support showed small negative correlations at T5, T7, and T8. It is possible that other, unmeasured family-related factors—such as parental attitudes toward mental-health care, perceived family burden, or stigma surrounding mental illness—are more pertinent to service use. Parental attitudes strongly shape help-seeking: negative views or stigma can discourage families from accessing services (Platell et al., 2023), and family burden may either motivate or deter service use depending on the level of stress involved (Gümüş & Kaçan, 2023; Tsang et al., 2020). Third, family dynamics can shift rapidly (Repetti et al., 2009; Verbruggen et al., 2020). We collected data biennially; therefore, the study may not have captured these short-term fluctuations. Consequently, changes in the family environment that occurred between assessments—changes that could influence adolescents’ help-seeking—may have been missed. Lastly, relatively few adolescents in the sample used mental-health services, which may have reduced the statistical power to detect small effects.

Consistent with previous research (Reeder et al., 2020; Reigstad et al., 2006; Ryan et al., 2014), prior use of mental-health services strongly predicts subsequent utilisation. This pattern corresponds with evidence that many psychiatric conditions persist across childhood and adolescence (Jansen et al., 2013) and that once an adolescent is engaged in care, follow-up or long-term management often continues (Janicke et al., 2001). For some youths, ongoing contacts address the same unresolved symptoms, indicating that standard interventions do not always achieve full remission (Brattfjell et al., 2023). For others, conditions such as ADHD are managed rather than “cured,” making sustained or recurrent service use expected (Faraone et al., 2024), hence long-term or recurring use of mental health services would be expected. Thus, the stability in specialist service use observed here likely reflects adolescents’ continuing need for support as they navigate developmental challenges—such as school transitions and peer difficulties—associated with their diagnoses. This continuity and increasing prevalence over time is also consistent with national statistics showing that demand for specialist child and adolescent mental health services remained high during the COVID‑19 period, with referrals increasing by about 15% from 2019 to 2021 and many municipalities reporting further increases in 2022 (Ministry of Health and Care Services, 2023), which coincides with the period when the 16- and 18-year follow-up data were collected.

### Limitations

Although the study has several strengths—namely, the use of a community sample, four biennial follow-ups spanning a broad age range, interview-based assessments of service use and symptoms, and statistical control for numerous confounders—several limitations should be acknowledged. First, we assessed whether the adolescents had used specialty mental-health service or not; future research should also examine facets such as service quality, duration, and treatment outcomes. Second, because the parents were relatively well educated, the findings may not generalize to less advantaged populations. Generalizability is further constrained by Norway’s publicly funded, universal health-care system, which differs from systems with private insurance or limited access (Øvretveit, 2003). Third, data were collected at two-year intervals. While this design captures long-term developmental change, more frequent assessments might detect short-term associations between family factors and help-seeking. Finally, although interviewer-administered measures reduce recall bias, both parent and adolescent reports remain vulnerable to memory errors and reluctance to disclose sensitive information. Incorporating additional data sources—such as administrative health records—could strengthen future investigations.

## Conclusion

Family factors are widely regarded as critical for facilitating adolescents’ access to mental-health services by fostering supportive environments, encouraging early recognition of distress, initiating help-seeking behaviors, and acting as gatekeepers for service use. However, the present study found no evidence that family-enabling variables—family functioning, social support, and interparental conflict—predicted Norwegian adolescents’ use of specialty mental-health services.

## Supplementary Information

Below is the link to the electronic supplementary material.

Supplementary Material 1 (DOCX 158 KB) 

## Data Availability

The data cannot be made available due to consent restrictions from the participants.

## Code Availability

The syntax from the data analyses is available upon request.
