# Supplementary material for: Family Predictors of Specialty Mental Health Service Use in Adolescents: A Prospective Cohort Study
Source: Res Child Adolesc Psychopathol. 2026 Jan 7;54(1):3. doi: 10.1007/s10802-025-01411-0 (PMC12775090; doi:10.1007/s10802-025-01411-0)
Supplement: Supplementary file 1 — Supplementary Material 1 (DOCX 158 KB) [file 10802_2025_1411_MOESM1_ESM.docx]

**Figure S1.**

*Trondheim Early Secure Study (TESS), Procedure and flow of participants*
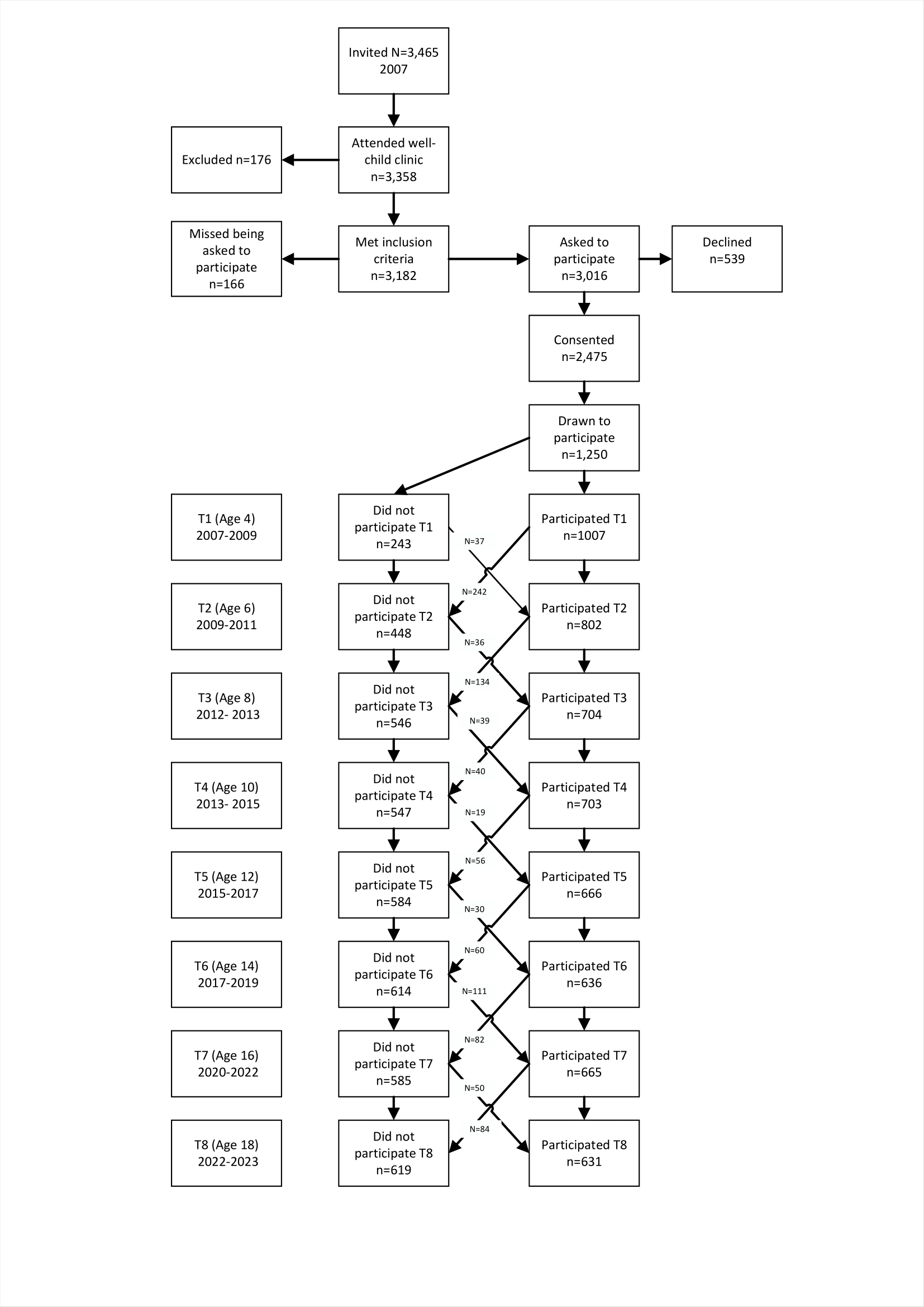


Note: T1 = Time 1, T2 = Time 2, etc. Participant numbers at each assessment point reflect the number of individuals invited to participate minus those who did not participate at the respective time points.

***Table S1.***

*Bivariate correlations between family factors and specialty service use*

|  | Service use (T5) | Service use (T6) | Service use (T7) | Service use (T8) |
| --- | --- | --- | --- | --- |
| Family functioning (T5) | -.06 | -.02 | .02 | -.04 |
| Family functioning (T6) |  | .03 | .05 | -.03 |
| Family functioning (T7) |  |  | -.05 | -.09 |
| Family functioning (T8) |  |  |  | -0.07 |
| Social support (T5) | -.11* | -.06 | -.03 | -.09 |
| Social support (T6) |  | -.01 | -.07 | -.01 |
| Social support (T7) |  |  | -.11* | -.09 |
| Social support (T8) |  |  |  | -.10* |
| Interparental conflict (T5) | -.01 | .05 | .03 | .07 |
| Interparental conflict (T6) |  | .01 | -.02 | .00 |
| Interparental conflict (T7) |  |  | -.01 | .03 |
| Interparental conflict (T8) |  |  |  | .08 |

Note: Asterisks indicate level of statistical significance: * *p* ≤ 0.05, ** *p* ≤ 0.01, ****p* ≤ 0.001.

***Table S2.***

*Standardized autoregressive paths for predictors across adjacent time points*

|  | 14 years | | | 16 years | | | 18 years | | |
| --- | --- | --- | --- | --- | --- | --- | --- | --- | --- |
|  | β | 95% CI | *p* | β | 95% CI | *p* | β | 95% CI | *p* |
| Propensity score as covariate | 0.29 | (0.17, 0.40) | < .001 | 0.27 | (0.16, 0.38) | < .001 | 0.38 | (0.24, 0.52) | < .001 |
| Family functioning | 0.62 | (0.54, 0.70) | < .001 | 0.28 | (0.13, 0.43) | < .001 | 0.43 | (0.31, 0.55) | < .001 |
| Social support | 0.35 | (0.28, 0.43) | < .001 | 0.37 | (0.31, 0.44) | < .001 | 0.36 | (0.29, 0.43) | < .001 |
| Interparental sonflict | 0.66 | (0.58, 0.74) | < .001 | 0.57 | (0.50, 0.64) | < .001 | 0.57 | (0.50, 0.65) | < .001 |

*Note.* Values represent standardized coefficients and 95% confidence intervals, and exact *p*-values.

***Table S3.***

*Standardized concurrent covariances between predictors and specialty service use at the same time point*

| Predictor  (same wave) | Service use  (12 years) | | Service use  (14 years) | | Service use  (16 years) | | Service use  (18 years) | |
| --- | --- | --- | --- | --- | --- | --- | --- | --- |
|  | Cov (SE) | *p* | Cov (SE) | *p* | Cov (SE) | *p* | Cov (SE) | *p* |
| Propensity score | 0.56 (0.09) | < .001 | 0.34 (0.10) | .005 | 0.52 (0.06) | < .001 | 0.32 (0.07) | < .001 |
| Family functioning | -0.06 (0.04) | .11 | 0.07 (0.04) | .11 | -0.06 (0.05) | .27 | -0.03 (0.04) | .47 |
| Social support | -0.11 (0.04) | .02 | 0.04 (0.04) | .38 | -0.08 (0.04) | .05 | -0.03 (0.04) | .42 |
| Interparental conflict | -0.02 (0.05) | .60 | -0.05 (0.06) | .47 | -0.03 (0.05) | .52 | 0.07 (0.05) | .16 |

*Note.* Values represent standardized covariances (standard errors in parentheses) between predictors and service use measured at the same time point.
